# Supplementary figures and images for: In vivo mapping of sodium homeostasis disturbances in individual ALS patients: A brain 23Na MRI study
Source: PLoS One. 2025 Jan 22;20(1):e0316916. doi: 10.1371/journal.pone.0316916 (PMC11753670; doi:10.1371/journal.pone.0316916)

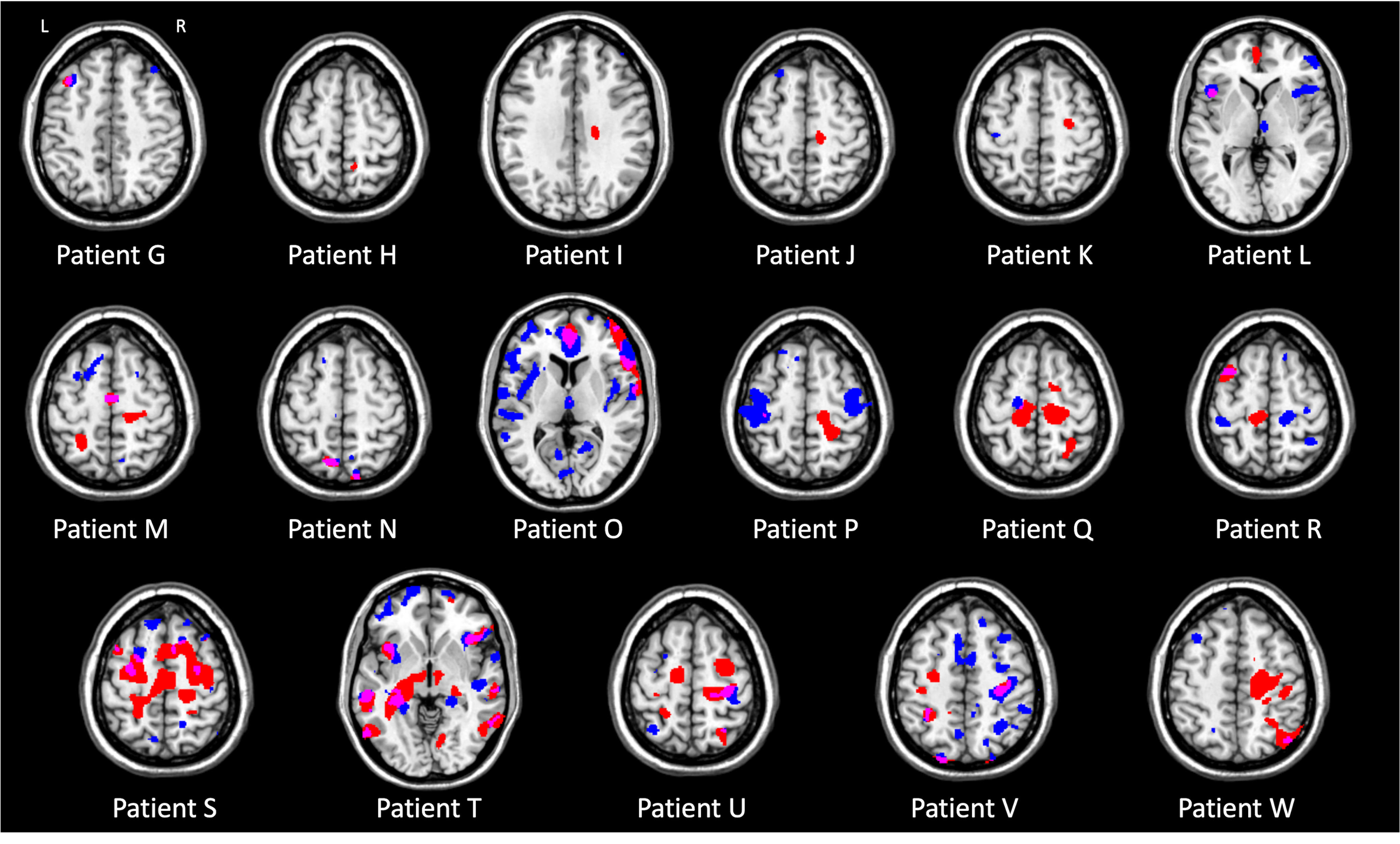

Supplement: S1 Fig — For each individual map, voxels with total sodium concentration (TSC) increase are represented in red and atrophy in blue. TSC increase can be related to atrophy in some patients as shown by the overlap between TSC increase and atrophy represented in pink. (TIF) [file pone.0316916.s001.tif]
