# Supplementary material for: In vivo mapping of sodium homeostasis disturbances in individual ALS patients: A brain 23Na MRI study
Source: PLoS One. 2025 Jan 22;20(1):e0316916. doi: 10.1371/journal.pone.0316916 (PMC11753670; doi:10.1371/journal.pone.0316916)
Supplement: Attachment — Submitted filename: PONE-D-24-27226_review-comments.docx [file pone.0316916.s003.docx]

**Review of Paper PONE-D-24-27226**

**Title:**

**In vivo mapping of sodium homeostasis disturbances in individual ALS patients: a brain 23Na MRI study**

**Summary:**

The purpose of this study was to investigate the severity of ALS at the individual level using brain 23Na MRIs in ALS patients. The study used ${}^{23}{Na}$MRI to map sodium homeostasis disturbances in ALS patients, revealing that the increase in total sodium concentration (TSC) varies among individuals. This variation is linked to clinical presentation and disease severity. The findings suggest that TSC increase could serve as a useful marker for the clinical heterogeneity in ALS, which could significantly impact the outcomes of therapeutic trials.

**Strengths And Weaknesses:**

## Strengths

- It has a thorough explanation of the main idea.
- Clearly explain the methodology.
- As a case study it has a good experimental design.

## Weaknesses

Overall, the writing can be significantly improved to address the following concerns.

- **Introduction:** You can add more references in the related work to show that you check new works.
- **Methodology:**
  - Put Figures in the right place.
  - You didn’t define a clear biomarker based on your finding
  - Normalize rate for each subject

**Questions:**

Answering the following questions and addressing the weaknesses above can significantly improve my score.

1. How can you include the effect of normalization rate foe each subject?
2. What is your suggestion for future works?
